# Expression of NUMB Protein and Its Prognostic Significance in Endometrial Cancer: A Retrospective Cohort Study

**DOI:** 10.7759/cureus.81402

**Published:** 2025-03-29

**Authors:** Vasileios Kartsiounis, Soultana Meditskou, Antonios Siargkas, Stavroula Pervana, Elisavet Pazarli, Ioannis A Kalogiannidis, Dimitrios Zouzoulas, Dimitrios Tsolakidis, Alexios Papanikolaou, Konstantinos Dinas

**Affiliations:** 1 3rd Department of Obstetrics and Gynecology, Aristotle University of Thessaloniki, Ippokrateio General Hospital, Thessaloniki, GRC; 2 Department of Histology and Embryology, Aristotle University of Thessaloniki, Thessaloniki, GRC; 3 Department of Obstetrics and Gynecology, General Hospital of Serres, Serres, GRC; 4 Department of Pathology, Papageorgiou General Hospital, Thessaloniki, GRC; 5 1st Department of Obstetrics and Gynecology, Aristotle University of Thessaloniki, Papageorgiou General Hospital, Thessaloniki, GRC; 6 2nd Department of Obstetrics and Gynecology, Aristotle University of Thessaloniki, Ippokrateio General Hospital, Thessaloniki, GRC

**Keywords:** clinicopathological correlation, endometrial cancer, molecular classification, numb protein, oncogenic pathways, prognostic biomarker, recurrence risk, survival analysis, tumor grade

## Abstract

Background and objectives

Endometrial cancer, the most prevalent gynecologic malignancy in developed countries, poses a major public health concern. NUMB, a multifunctional protein, has been implicated in the tumorigenesis of endometrial cancer. The goal of this study was to investigate the association of NUMB protein expression with clinicopathological characteristics and prognostic outcomes in patients with endometrial cancer.

Methods

A retrospective cohort study was performed on 41 patients with endometrial cancer who were divided into three groups according to the expression level of NUMB protein using immunohistochemistry: NUMB 1 (mild), NUMB 2 (moderate), and NUMB 3 (significant). Statistical analyses, including ANOVA, Kruskal-Wallis test, chi-squared test, Kaplan-Meier survival analysis, Cox proportional hazards model, and regression analyses, were used to assess the relationship between NUMB expression and survival as well as various clinicopathological parameters.

Results

Significant NUMB expression (NUMB 3) was found to be correlated with more aggressive clinicopathological characteristics, including higher tumor grade (p = 0.039), larger tumor size (p = 0.016), and elevated recurrence rate (p = 0.033). Patients with NUMB 3 expression also exhibited significantly poorer overall survival (p = 0.003) and had a 10-fold higher mortality risk compared to those with mild NUMB expression (HR: 9.67, p = 0.034) during the 10-year follow-up period.

Conclusion

This study demonstrates that significant NUMB protein expression is associated with aggressive clinicopathological features and poor prognostic outcomes in endometrial cancer. NUMB may serve as a potential prognostic biomarker for this disease. Further research is warranted to elucidate the precise role of NUMB in endometrial carcinogenesis and its therapeutic implications.

## Introduction

Endometrial cancer is the most common gynecologic malignancy in the developed world. It is a disease with complex etiology and significant public health implications [1].

In 2013, the Cancer Genome Atlas (TCGA) analyzed the genome of 373 patients with endometrial cancer and produced a new molecular classification system that is based on genetic alterations rather than histological findings. The new classification system is based on polymerase E gene (POLE), microsatellite instability (MSI), and chromosomal copy number abnormalities. Tumors are categorized into four molecular subgroups: POLE ultramutated, MSI hypermutated, genome copy-number low, and genome copy-number high [2].

Endometrial cancer encompasses various histological subtypes, each with distinct molecular characteristics. Endometrioid carcinomas frequently harbor PTEN mutations, affecting the PI3K/AKT/mTOR pathway and promoting uncontrolled cell growth. These tumors are typically microsatellite stable [3]. In contrast, serous carcinomas are characterized by prevalent p53 mutations, disrupting cell cycle regulation and DNA repair. They often express markers such as p16, IMP3, and HMGA2, with occasional ERBB2 overexpression, whereas abnormal staining for PTEN, β-catenin, ARID1A, and mismatch repair proteins is rare [4]. Clear cell carcinomas are commonly positive for HNF1β, napsin A, and AMACR (P504S), with p53 mutant immunostaining present in 22-72% of cases. Estrogen (ER) and progesterone receptors (PR) are typically negative or weakly positive [5-7].

NUMB is a gene on chromosome 14 that encodes the NUMB protein, which regulates key cellular processes, such as differentiation, growth, adhesion, and migration. It plays a crucial role in the Notch and Hedgehog signaling pathways, both involved in embryogenesis and carcinogenesis. The Hedgehog pathway regulates tissue patterning through interactions with the transmembrane protein Patch, activating Gli transcription factors, while NUMB facilitates their degradation via E3-ubiquitination ligase. The Notch pathway has been implicated in several cancers, including basal cell carcinoma, medulloblastoma, and malignancies of the pancreas, breast, colon, lung, and ovary [8,9].

In endometrial cancer, it appears to function as a tumor suppressor via its interaction with the Notch pathway, as mentioned previously, but also by stabilizing p53, preventing its degradation by MDM2 [9-11]. There is also evidence of elevated expression in endometrial cancer tissues compared to non-cancerous endometrial tissues [10]. In vitro research has also shown that NUMB overexpression induces apoptosis in endometrial cancer cells, whereas its knockdown increases their proliferation [10].

The aim of the present study is to correlate the expression of NUMB protein with clinical, histopathological parameters, and prognosis in endometrial cancer.

## Materials and methods

Study population

This retrospective cohort study included women diagnosed with endometrial cancer. Patients who had hysterectomy for endometrial cancer and had paraffin-embedded tissue blocks available were included. Patients with other synchronous malignancies or mixed histological types were excluded. Pathology specimens were retrieved from the Pathology Department of a tertiary university cancer center in Thessaloniki, Greece, between 2012 and 2016.

Our population was selected from a list of eligible endometrial cancer cases diagnosed and treated during the above period. Each case was assigned a unique number, and a computer-generated randomization was used to select the patients. The sample size for this study was calculated through a power analysis based on the primary 10-year survival outcome to have enough statistical power to detect associations between NUMB staining and long term survival. Based on this analysis a sample size of 40 was considered adequate for the primary analysis.

Patient demographic data, including age, height, and weight, were collected electronically and verified via telephone interviews when possible. Medical data, including operation type, preoperative CA-125 levels, pathology findings (macroscopic tumor type and size, histological type, tumor grade, FIGO staging), recurrence, and survival status, were extracted from the electronic database of the hospital. The study was approved by the Bioethics and Ethics Committee, Department of Medicine, Aristotle University of Thessaloniki, Greece, which issued approval number 1.695/20/10/2020, and written consent was obtained.

Immunohistochemical evaluation

For NUMB protein expression assessment, formalin-fixed, paraffin-embedded tissue sections were used. Representative paraffin blocks from hysterectomy specimens of eligible cases were processed to obtain 3-µm-thick sections, which were mounted on positively charged glass slides. After incubation at 58°C, Abcam® monoclonal antibody NUMB (EPR21988) was applied, and preliminary immunohistochemical testing determined the optimal antibody dilution (1:1000).

Immunohistochemical staining for NUMB was performed using the Streptavidin-Biotin Complex Method with the Autostainer Link 48 (Dako®). Hematoxylin counterstaining was applied, and semiquantitative immunohistochemical analysis was conducted following Soslow et al.'s method [12]. NUMB protein expression was graded based on the percentage of immunoreactive cells (staining density) and staining intensity. Staining density was rated as follows: 0%-10% of cells demonstrating staining scored 1; 11%-50% scored 2; 51%-80% scored 3; and 81%-100% scored 4. Staining intensity was rated on a scale of 0-3 as follows: 0 = negative; 1 = weak; 2 =moderate; and 3 = strong.

**Figure 1:**
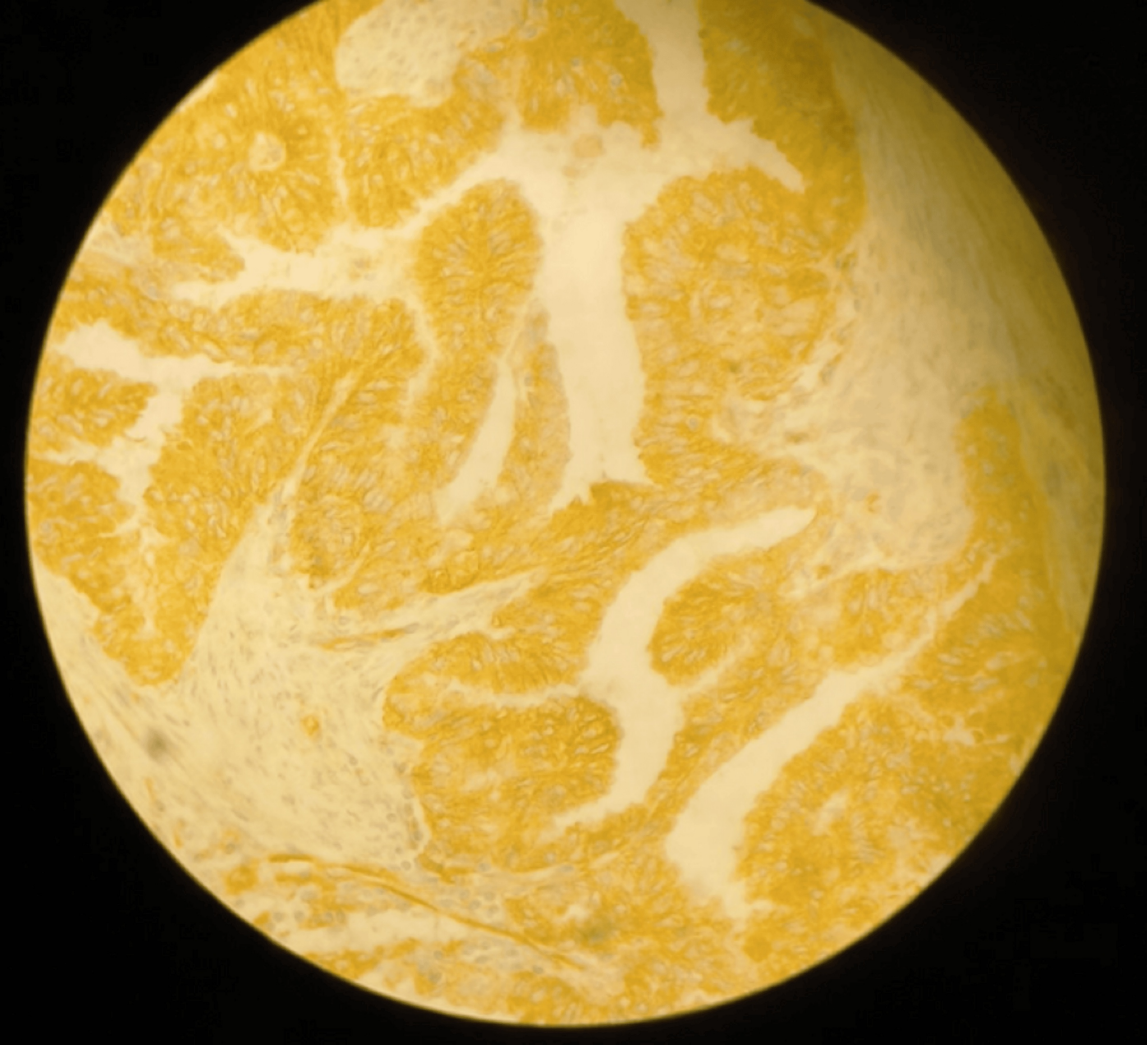
NUMB grade 2 (moderate) staining (microscope image)

The final immunohistochemical score (0-12) was calculated by multiplying density and intensity scores, forming the following grading scale: score 0 = No antibody expression; scores 1-3 = Grade 1 (mild); scores 4-6 = Grade 2 (moderate); scores 7-9 = Grade 3 (significant); and scores 10-12 = Grade 4 (strong) (Figure 1).

Study groups were compared according to NUMB expression grade (1, 2, and 3), as no cases with score 0 or 4 were observed. Comparisons across NUMB groups and population characteristics were performed to identify significant differences.

Outcomes

The primary outcome of this study was the association between NUMB expression and 10-year survival in patients with endometrial cancer. Given that endometrial cancer generally exhibits a less aggressive clinical course, a 10-year follow-up period was chosen to provide a comprehensive assessment of long-term survival. Survival time was analyzed using time-to-event data, measured in months from the date of diagnosis until the occurrence of death or the last recorded follow-up. Censoring was applied to patients who were alive at the end of the follow-up period to account for incomplete survival observations.

Secondary outcomes examined the association between NUMB expression and various clinicoepidemiological and pathological parameters, including age, BMI, preoperative serum CA-125 levels, tumor size, tumor grade, the International Federation of Gynecology and Obstetrics (FIGO) stage, and tumor recurrence.

Statistical analysis

Descriptive statistics were calculated to summarize the characteristics of the study population. The distribution of continuous variables was assessed using the Shapiro-Wilk test. Age, BMI, weight, and height were approximately normally distributed and are presented as mean ± standard deviation (SD). CA-125 levels and tumor maximum diameter values were not normally distributed and are presented as median and interquartile range (IQR). Categorical variables are presented as counts and percentages.

For continuous variables with normal distributions, one-way analysis of variance (ANOVA) was used to compare means across the groups. For continuous variables with non-normal distributions, the Kruskal-Wallis test was employed to compare medians. Categorical variables were compared using the chi-squared test or Fisher's exact test when expected cell counts were less than five. The statistically significant associations were further explored by utilizing logistic and linear regressions in order to calculate specific effect measures, odds ratio (OR), and their 95% confidence intervals (CI).

Kaplan-Meier survival analysis was performed to estimate survival probabilities for each NUMB group, and survival curves were compared using the log-rank test. The Cox proportional hazards model was used to estimate hazard ratios (HR) with 95% CI in order to assess the association of NUMB expression with survival and independent clinical and pathological variables.

All analyses were performed in R (Development Core Team, Vienna, Austria) using the R basic stats and survival package. Results were considered statistically significant if the two-sided p-value was below 0.05.

## Results

In the studied population of 41 cases, statistical examination discovered strong correlations between levels of NUMB expression and multiple clinical and pathological features. NUMB 1, NUMB 2, and NUMB 3 were found in 39%, 24.4%, and 36.6% of cases, respectively. No significant differences were observed in age, BMI, weight, or preoperative CA-125 levels. NUMB 3 was more likely to be associated with high-grade tumors, with 46.7% of cases categorized as grade 3 tumors, while NUMB 1 was more common in grade 1 tumors (43.8%) (p = 0.039). NUMB 3 was further associated with type 2 tumors (46.7%), whereas NUMB 1 was only found in type 1 tumors (100%) (p = 0.030). Tumor size differed broadly across groups, with NUMB 3 having the largest median diameter (4.00 cm; 3.00-5.00 IQR; p = 0.016). There were also differences in clinical outcomes among groups. Recurrence in NUMB 3 was higher at 33.3% than in NUMB 1 (6.2%) (p = 0.033), and mortality was significantly greater in NUMB 3 (60%) than in NUMB 1 (6.2%) and NUMB 2 (20%) (p = 0.003) (Table 1).

**Table 1:** Association between NUMB levels and clinicopathological characteristics or adverse outcomes Abbreviations: IQR = interquartile range; n = number of cases; SD = standard deviation; FIGO = International Federation of Gynecology and Obstetrics Normal continuous variables were analyzed using one-way analysis of variance, with the F-statistic reported. Non-normal continuous variables were assessed with the Kruskal-Wallis test, with the H-statistic reported. Categorical variables were analyzed using the chi-squared test, with the χ² statistic reported. A p-value of less than 0.05 was considered the threshold for statistical significance.

Items	Levels	Overall	NUMB 1	NUMB 2	NUMB 3	Statistic	P-value
N		41	16 (39%)	10 (24.4%)	15 (36.6%)		
Age (years), mean ± SD		64.80 ± 9.60	62.75 ± 9.02	66.30 ± 11.36	66.00 ± 9.22	0.59–F	0.558
FIGO stage, n (%)	I	32 (78.0)	16 (100.0)	8 (80.0)	8 (53.3)	12.53–χ²	0.051
	II	3 (7.3)	0 (0.0)	1 (10.0)	2 (13.3)		
	III	2 (4.9)	0 (0.0)	1 (10.0)	1 (6.7)		
	IV	4 (9.8)	0 (0.0)	0 (0.0)	4 (26.7)		
Grade, n (%)	1	13 (31.7)	7 (43.8)	2 (20.0)	4 (26.7)	10.06–χ²	0.039
	2	17 (41.5)	9 (56.2)	4 (40.0)	4 (26.7)		
	3	11 (26.8)	0 (0.0)	4 (40.0)	7 (46.7)		
CA-125 (U/mL), median (IQR)		14.50 (10.35, 24.18)	13.30 (9.60, 15.90)	25.45 (22.05, 27.58)	13.10 (9.60, 13.70)	3.68–Η	0.158
Type, n (%)	1	30 (73.2)	16 (100)	6 (60.0)	8 (53.3)	9.76–χ²	0.008
	2	11 (26.8)	0 (0.0)	4 (40.0)	7 (46.7)		
Tumor maximum diameter (cm), median (IQR)		3.00 (2.00, 4.00)	2.20 (2.00, 3.00)	4.00 (2.50, 4.00)	4.00 (3.00, 5.00)	8.23–Η	0.016
Recurrence, n (%)		6 (14.6)	1 (6.2)	0 (0.0)	5 (33.3)	6.81–χ²	0.033
Weight (kg), mean ± SD		86.83 ± 18.98	91.66 ± 19.31	78.80 ± 20.25	87.04 ± 16.99	0.249–F	0.249
Height (m), mean ± SD		1.62 ± 0.07	1.63 ± 0.06	1.57 ± 0.07	1.64 ± 0.05	0.029–F	0.029
BMI (kg/m^2^), mean ± SD		33.12 ± 6.95	34.36 ± 6.22	31.94 ± 8.54	32.54 ± 6.82	0.651–F	0.651
Death, n (%)		12 (29.3)	1 (6.2)	2 (20.0)	9 (60.0)	11.35–χ²	0.003

Survival analysis

**Figure 2:**
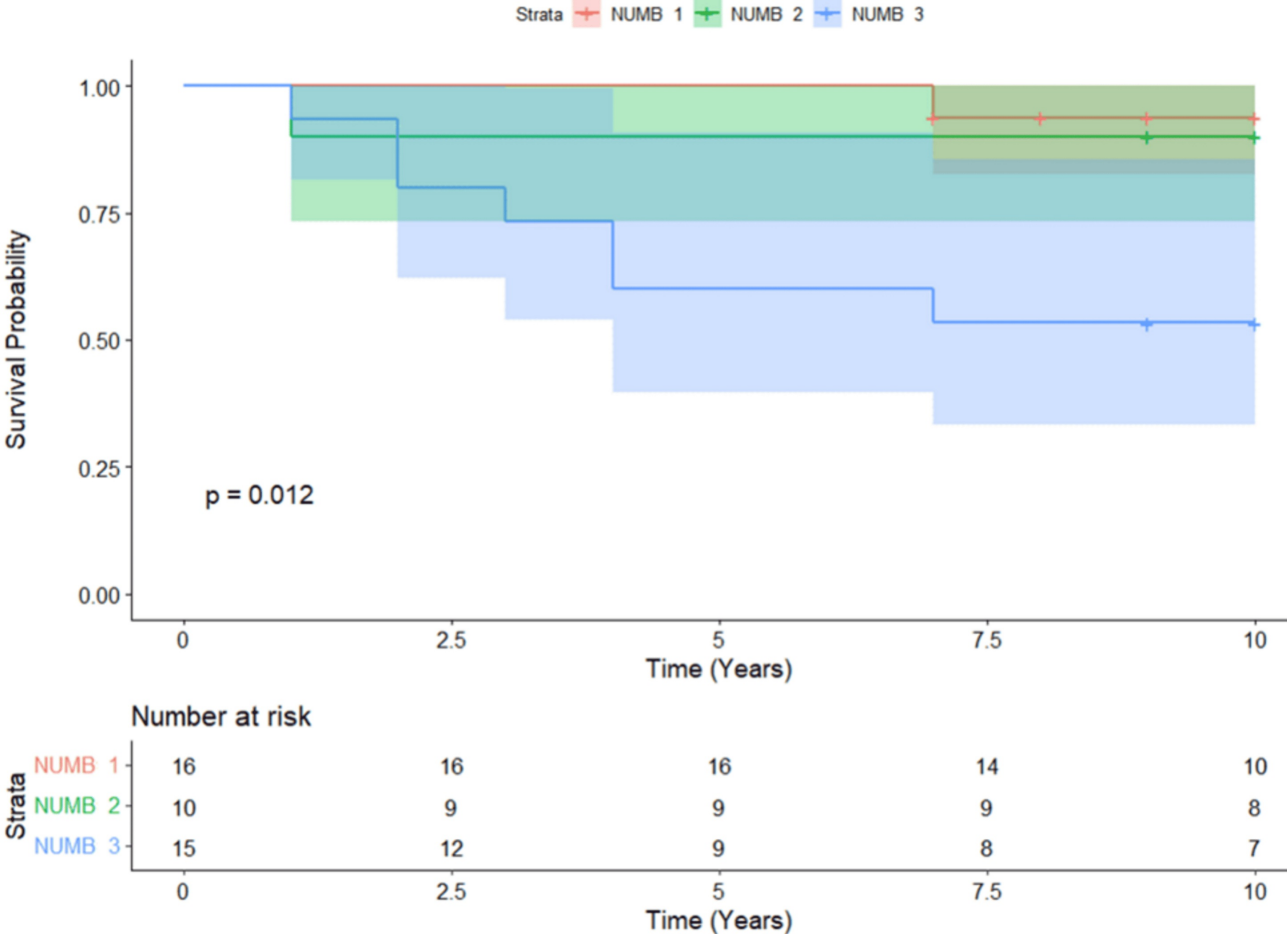
Kaplan Meier 10-year survival analysis for NUMB scores

There was a significant difference between the survival probabilities over time among the different NUMB groups, according to Kaplan-Meier survival analysis (log-rank test, p = 0.012) (Figure 2). According to Cox proportional hazards model analysis, patients with NUMB 3 had a 10-fold higher risk of death compared to NUMB 1 (HR: 9.67, 95% CI: 1.19-78.9, p = 0.034). However, there was no significant association with NUMB 2 patients (HR: 1.74, 95% CI: 0.109-27.9, p = 0.694) (Table 2).

**Table 2:** Cox proportional hazards model comparing NUMB 1 (control group) with NUMB 2 and 3 (study groups), assessing 10-year survival Abbreviations: CI = Confidence intervals. Cox proportional hazards model comparing NUMB 1 (control group) with NUMB 2 and 3 (study groups). A p-value of less than 0.05 was considered the threshold for statistical significance.

Items	Hazard Ratio	95% CI	P-value
NUMB 2	1.74	0.11, 27.90	0.694
NUMB 3	9.67	1.19, 78.90	0.034

Regression analyses

Regression analysis further described the association between NUMB expression and major clinical and pathological outcomes. The odds of death were 22 times higher in NUMB 3 patients compared to patients with NUMB 1 (OR: 22.50, 95% CI: 2.32-218.35, p = 0.007). While NUMB 3 correlated with increased recurrence risk (OR: 7.50, 95% CI: 0.76-74.16), this did not reach statistical significance (p = 0.085). Aside from its link to clinical outcomes, NUMB expression was also correlated with tumor size, a pathological parameter. NUMB 3 was strongly associated with bigger lesions with an estimated 1.72 cm larger maximum tumor diameter when compared to NUMB 1 lesions (95% CI: 0.59-2.84, p = 0.004). However, such association was not statistically significant with NUMB 2 and lesion size (OR: 1.13, 95% CI: -0.08-2.34, p = 0.067) (Table 3).

**Table 3:** Association of NUMB scores with mortality, recurrence, and tumor size Abbreviations: CI = Confidence intervals. Logistic/linear regressions were employed. A p-value of less than 0.05 was considered the threshold for statistical significance.

Variables	Odds Ratio	95% CI	P-value
NUMB 2 - death	3.75	0.29, 47.99	0.310
NUMB 2 - recurrence	0.00	0.00, +∞	0.996
NUMB 2 - tumor maximum diameter (cm)	1.13	-0.08, 2.34	0.067
NUMB 3 - death	22.50	2.32, 218.35	0.007
NUMB 3 - recurrence	7.50	0.76, 74.16	0.085
NUMB 3 - tumor maximum diameter (cm)	1.72	0.59, 2.84	0.004

**Figure 3:**
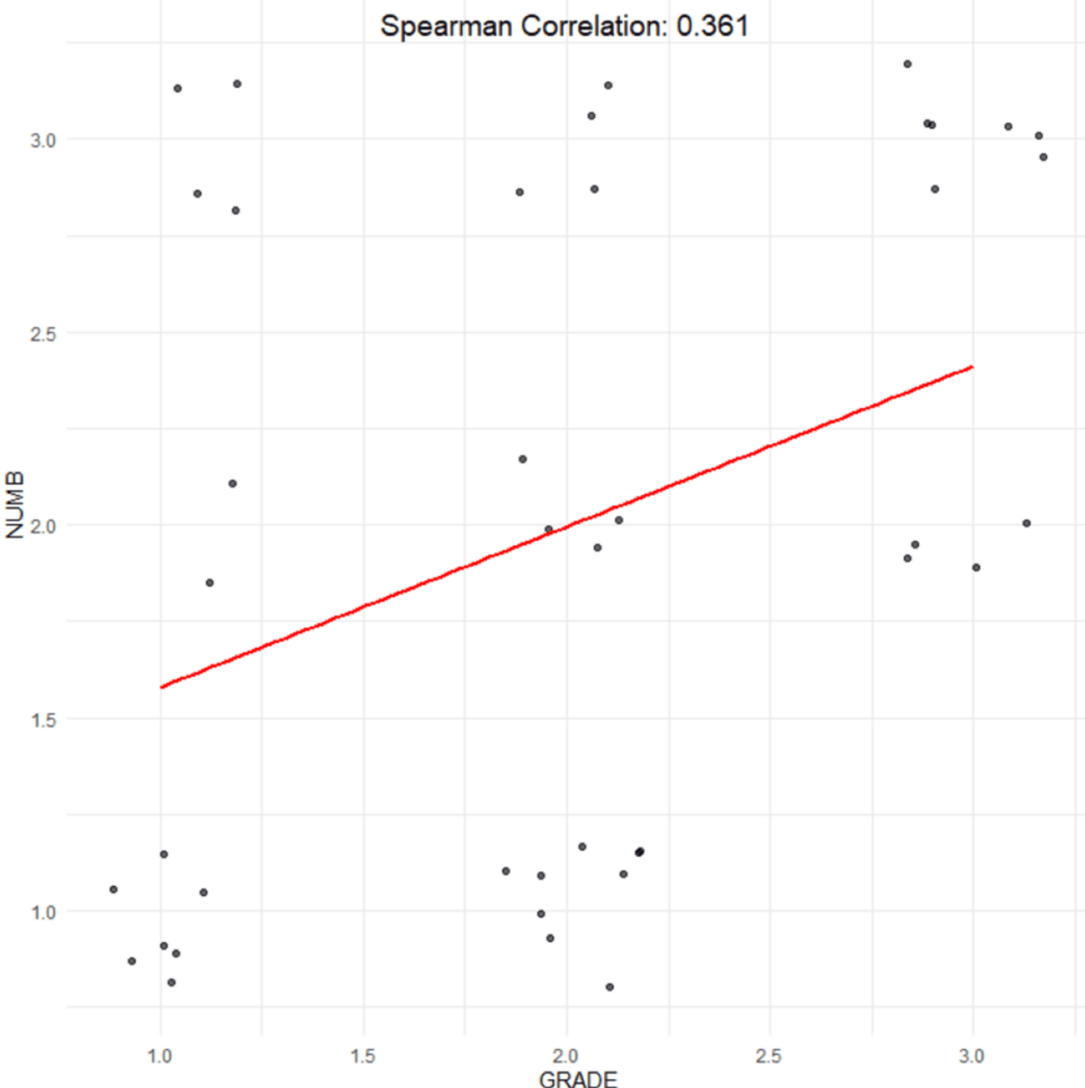
Depiction of the linear relationship between NUMB scores and cancer grade

Additionally, Spearman's correlation analysis demonstrated a positive correlation between NUMB expression and tumor grade (rho = 0.361, p = 0.020) (Figure 3).

## Discussion

Principal findings

This study demonstrates that significant NUMB expression (NUMB 3) is strongly associated with adverse clinicopathological features in endometrial cancer. Specifically, patients with NUMB 3 had a markedly higher hazard of death, greater odds of recurrence, larger tumor size, and higher tumor grade compared to those with lower NUMB expression (NUMB 1). These findings suggest that elevated NUMB protein expression correlates with more aggressive tumor behavior and poorer long-term outcomes.

Interpretation of the findings

Patients with NUMB 3 had a 10-fold increased risk of death compared to those with NUMB 1 (HR: 9.67, 95% CI: 1.19-78.9, p = 0.034), while NUMB 2 was not significantly associated with increased mortality (HR: 1.74, 95% CI: 0.109-27.9, p = 0.694). The odds of death were 22 times higher in the NUMB 3 group compared to NUMB 1 (OR: 22.50, 95% CI: 2.32-218.35, p = 0.007).

Regarding recurrence, initial analysis revealed a significant difference across NUMB groups, with NUMB 3 having a recurrence prevalence of 33.3% compared to 6.2% in NUMB 1. Logistic regression showed increased odds for recurrence in NUMB 3 patients (OR: 7.50, 95% CI: 0.76-74.16), though statistical significance was not reached (p = 0.085), likely due to the limited sample size. The association between NUMB expression and recurrence is consistent with the observation that NUMB 3 patients also exhibited higher tumor grade, as confirmed by both chi-squared analysis and Spearman correlation.

Beyond clinical outcomes, NUMB 3 was significantly associated with larger tumors, with an estimated 1.72 cm increase in lesion diameter compared to NUMB 1 (95% CI: 0.59-2.84, p = 0.004). This reinforces its potential role in disease progression. Conversely, NUMB 2 did not show a significant association with tumor size (OR: 1.13, 95% CI: -0.08-2.34, p = 0.067).

Comparison with existing literature

NUMB has been implicated as a tumor suppressor in various cancers by inhibiting Notch signaling and promoting p53 stabilization [8]. Previous studies have shown that NUMB expression is elevated in endometrial cancer tissues compared to normal tissues and is predominantly localized in the nucleus, with some presence in the plasma membrane [10,11]. Experimental evidence further suggests that NUMB overexpression induces apoptosis in Ishikawa endometrial cancer cells, whereas NUMB inhibition enhances cell proliferation, and there is evidence that it acts as a tumor suppressor [10].

A study using the Cancer Genome Atlas (TCGA) examined NUMB and its homolog NUMBL across multiple tumor types, including endometrial cancer, and found that NUMBL expression was associated with poorer prognosis [13]. However, that study focused primarily on NUMBL and applied genomic rather than protein expression analyses, differing from this study, which directly evaluated NUMB protein expression and its clinicopathological correlations.

In ovarian cancer, it suppresses proliferation and epithelial-mesenchymal transition (EMT) via the p21-activated kinase 1 (PAK1)/β-catenin pathway [14], while its knockdown enhances cisplatin sensitivity in ovarian cancer cells by inhibiting proliferation and EMT [15]. Similar tumor-suppressive properties have been observed in cervical cancer, where Interleukin-8 (IL-8) has been shown to downregulate NUMB expression while upregulating IL-8 receptors and extracellular signal-regulated kinase (ERK) signaling, indicating a role in tumor proliferation and migration [16]. In lung cancer, there is some evidence that it may function as an oncogene via alternative splicing when exon 9 is included [17].

NUMB interacts with several oncogenic pathways (Notch, Hedgehog, PAK1/β-catenin, and wingless/integrated (Wnt)/β-catenin pathways), all suggested to be involved in endometrial carcinogenesis [8,18]. NUMB promotes degradation of the Gli transcription factors in the Hedgehog pathway that mediate effects on proliferation and differentiation [9]. The PAK1/β-catenin pathway, demonstrated in ovarian cancer [14], influences EMT by modulating β-catenin activity through cytoskeletal remodeling, while the Wnt/β-catenin pathway is a canonical signaling cascade implicated in cell proliferation and differentiation [8,18]. Moreover, loss of NUMB has been linked to aggressive bladder cancer, thereby suggesting its function as a tumor suppressor gene in different cancer types [19].

Translational applications of NUMB include its potential as a biomarker for risk stratification, given its correlation with survival [13]. NUMB knockdown has been shown to enhance cisplatin sensitivity in ovarian cancer cells by inhibiting proliferation and EMT [15]. Melatonin has been found to inhibit 17β-estradiol (E2)-induced proliferation, invasion, and EMT in endometrial adenocarcinoma cells by upregulating NUMB and E-cadherin, indicating a potential role for NUMB-targeting hormonal therapies [20]. Additionally, NUMB has been implicated in colorectal cancer progression, where its loss is associated with increased EMT activity and Wnt pathway activation [21]. These findings suggest that targeting NUMB-related pathways could improve treatment efficacy in multiple tumor types.

Over the last years, a rise in the incidence of endometrial cancer in women of reproductive age has been noted [22]. That means that there is an increasing population of patients who would benefit from fertility-sparing treatment with a multidisciplinary approach [23]. Selection criteria have traditionally been based on histology; however, the molecular classification also seems to play a role now, as there has been some research demonstrating that some alterations, such as PTEN and POLE, appear to be good prognostic factors [2,24,25]. It would be plausible to use molecular methods in order to select or exclude potential candidates for fertility-sparing treatment of endometrial cancer, and NUMB could also be the subject of oncofertility research in future studies.

Strengths and limitations

One of the major strengths of this study is its random case selection with rigid inclusion criteria and availability of reliable histopathological and clinical data. The 10-year follow-up period adds to the robustness of the survival estimates, and a power analysis showed that the sample size was adequately powered to detect primary survival outcomes.

Of course, the study is not without its limitations. The relatively small sample size also limited the statistical power to detect certain associations, particularly for recurrence, where a notable trend was observed, but it did not reach statistical significance. The study is also a single-center, retrospective study, which reflects a potential for selection bias. Additionally, there was no mechanistic investigation, leaving questions about NUMB's precise involvement in tumor progression unanswered. Finally, while some deaths in the cohort could be attributed to aging rather than disease progression, the observed links between NUMB expression, recurrence, and survival strongly indicate its prognostic significance.

## Conclusions

Significant levels of NUMB expression (NUMB 3) were associated with worse endometrial cancer outcomes, according to the findings of this study. Patients with elevated levels of NUMB appear to have more aggressive tumors, increased recurrence rates, larger tumors, and poorer long-term prognosis. As the field of oncology enters the era of molecular classification, NUMB may serve as a prognostic biomarker. However, to better understand the mechanisms driving tumorigenesis and tumor progression, further studies are needed.
